# Effects of intralesional pulsed radiofrequency treatment on pain in patients with calcaneal spur: results of 460 patients

**DOI:** 10.1186/s12891-021-04926-x

**Published:** 2021-12-10

**Authors:** Ibrahim Eke, Mehmet Akif Akcal, Ali Vefa Sayrac, Yusuf Iyetin

**Affiliations:** 1 Orthopaedics and Traumatology Specialist, Orthopaedics and Traumatology Department, Ataturk State Hospital, Ucgen Mah. Gulluk Cad. No:100, Antalya, Turkey; 2 Emergency Medicine Specialist, Emergency Department, Ataturk State Hospital, Antalya, Turkey; 3 Orthopaedics and Traumatology specialist, Orthopaedics and Traumatology Department, Private Pendik Regional Hospital, Istanbul, Turkey

**Keywords:** Heel pain, Calcaneal spur, Pulsed radiofrequency treatment

## Abstract

**Background:**

This study aimed to investigate the efficacy of intralesional pulsed radiofrequency (RF) in the treatment of calcaneal spur and the results of patients who underwent single and double sessions of RF treatment.

**Methods:**

The population of this retrospective study consisted of 460 patients who were diagnosed with calcaneal spur with clinical examination and direct radiography. The Wong-Baker Faces Pain Rating Scale and The American Orthopaedic Foot and Ankle Society (AOFAS) Ankle-Hindfoot Score were used to determine the pain status and functional capacities of the patients. Posttreatment evaluation was carried out on average in the 6th week.

**Results:**

The study involved 460 patients, 76.9% of whom were female, with the average age of 50.8 ± 10.9 years in total. Of the patients 43% was given RF therapy in a single session, and 57% of them in double sessions. After the RF procedure, the number of patients whose pain decreased according to both AOFAS and Wong-Baker pain scoring systems increased statistically significantly (*p* < 0.001). There was a statistically significant increase in the AOFAS-pain scores and the total AOFAS scores and a significant decrease in the Wong Baker-pain scale after treatment. However, there was no significant change in treatment success with respect to the number of RF sessions. Although not statistically significant, the differences in the AOFAS-pain scores and in the total AOFAS scores were found to be higher in patients who underwent single session RF, while the difference in the Wong Baker-pain ranking was higher in patients who received double sessions RF.

**Conclusion:**

Intralesional pulsed RF procedure can be preferred as a relatively less invasive method that does not have any serious complications in patients with persistent calcaneal spurs who do not respond to the use of oral anti-inflammatory drugs and shoe insoles, nor corticosteroid injection to the lesion area.

## Background

Heel pain is a very common health problem that is likely to influence people from all ages and reduce the quality of life [1]. Calcaneal spur, one of the most common causes of heel pain, is an outgrowth originating from the calcaneal tuberosity, and often presents itself as secondary to mechanical bone traction caused by plantar fasciitis [2–4]. The exact cause of the pain associated with the calcaneal spur is inflammation at the attachment site of the plantar fascia, which plays an important role in walking [5].

Despite the lack of clear information about the prevalence of heel spurs in the society or about which gender and age group is more affected, studies have shown that the incidence generally ranges from between 10 to 35%, being more common in women, with its frequency increasing with age [6–9].

Heel spurs are not only treated by conventional therapies such as non-steroidal anti-inflammatory drugs, shoe insoles (shoe pads), corticosteroid injection to the lesion area, orthoses, exercises, and night splints, but also by innovative treatment methods such as radiotherapy, extracorporeal shock waves, and percutaneous and/or intralesional radiofrequency application, which have also been preferred in recent years, especially in persistent and recurrent cases, whereas calcaneal spur excision is often regarded as the last option [3, 4, 10]. Treatment methods such as exercises, corticosteroid injections, excision of the calcaneal spur are aimed at the etiology and the biomechanical changes that occur while intralesional pulsed radiofrequency application is a completely symptomatic, pain-relieving treatment option. The most important goal in such therapies is to increase the quality of life by reducing pain.

There are studies showing that radiofrequency is successfully used in the treatment of numerous pain conditions such as radicular pain, trigeminal neuralgia, complex regional pain syndrome, sacroiliac joint pain, facet arthropathy, shoulder pain, chronic postsurgical pain, and myofascial pain apart from calcaneal spur [11, 12].

Thermal RF lesioning produces a lesion by causing destruction with heat and is formed by the passage of a very high frequency through the tissue [12, 13]. Although there are studies showing that pulsed RF has been used successfully in chronic pain conditions for the last 20–25 years, its mechanism of action has not been explained as clearly as continuous RF lesioning. An alteration in synaptic transmission is one of the hypotheses put forward [14], and another is the stimulation of the noradrenergic and serotonergic systems. The change in gene expression by neuromodulation has also been suggested as a mechanism explaining the effect of pulsed RF application [12]. On the other hand, it has been clearly demonstrated that pulsed RF does not cause nerve damage [13].

The medial calcaneal nerve and lateral plantar nerve are the main branches of the posterior tibial nerve, providing sensory innervation of the heel. By applying radiofrequency, it is aimed to treat pain by ablation of these nerves [3, 15, 16]. In the literature, there are studies showing that not only RF application on the nerves but also intraarticular or intralesional RF application is effective in pain treatment [17–20]. So, this study aimed to investigate the effectiveness of intralesional pulsed RF in the treatment of calcaneal spur by evaluating the results we obtained in a large series of patients.

## Methods

### Study design, participants and technique

The population of this retrospective study consisted of 571 patients who were diagnosed with calcaneal spur with clinical examination and direct radiography at the Orthopaedics Clinic at Ataturk Hospital, Antalya, Turkey between December 17, 2019 and October 17, 2020, and received radiofrequency therapy since no response had been achieved from varying therapies such as oral anti-inflammatory drugs, shoe insoles, and a single dose of corticosteroid injection to the lesion area. The patients whose files, radiological images and examination findings before and/or after treatment could not be reached were excluded from the study, which then continued with 460 patients. The Wong-Baker Faces Pain Rating Scale and AOFAS Ankle-Hindfoot Scale were used to determine the pain status and functional capacities of the patients. The patients were compared in terms of these scores before and after the treatment according to their demographic characteristics, the number of treatment sessions and body mass index (BMI). Post-treatment evaluation was carried out on average in the 4th week. Among the patients coming for control, a second session was recommended for those with ongoing pain, and the patients who accepted were treated. In the procedure, after the patients were placed in the prone position, asepsis was performed with polyvinyl pyrrolidone-iodine, and then the lesion and needle insertion sites were locally anesthetized with 1 ml, 2% lidocaine, and intralesional pulsed RF was applied with a BNF-RF generator for 360 s at 42 degrees. After the procedure, patients were followed-up for 1 h in case of any complications.

### Data collection

Demographic, radiographic and clinical characteristics of the patients in the study group, as well as their treatment and outcome data were obtained from electronic medical records and patients’ files.

### Ethical approval

Prior to the study, the necessary approval was obtained from the Clinical Research Ethics Committee of University of Health Sciences, Antalya Training and Research Hospital. Since it was a retrospective study, informed consent for inclusion in the study could not be obtained from the patients. But before the patients were given radiofrequency treatment, consent for treatment permission was obtained. The study was conducted in accordance with the Declaration of Helsinki.

### Statistical analysis

The descriptive findings were presented with mean ± standard deviation (SD) or median (min-max) for the continuous data, and with frequency and percentage for the categorical data. The normality assumptions were controlled by the Shapiro-Wilk test. Mann–Whitney U test was used for the analysis of non-normally distributed numerical data. McNemar-Bowker test was used to compare paired categorical data. Wilcoxon Signed Ranks test was used for the nonparametric comparison of repeated measurements. The relationship between BMI and pain scores was evaluated with the Spearman correlation test. Statistical analysis was made using IBM SPSS Statistics for Windows, Version 23.0 (IBM Corp., Armonk, NY). Two-sided *p* values < 0.05 were considered statistically significant.

## Results

The study involved 460 patients, 76.9% of whom were female, with the average age of 50.8 ± 10.9 years in total. Average BMI was calculated as 31.3 ± 5.1 kg/m^2^. The most frequent heel involvement was found to be on the right (51.5%). In the current study, 43% of the patients was given RF therapy in a single session, and 57% of them in double sessions. Detailed demographic, clinical, and treatment characteristics of the patients are given in Table 1 (Table 1).

**Table 1:** General demographic and treatment characteristics of all patients at the time of first evaluation

Variables	***n*** = 460
**Age (years), mean ± SD**	50.8 ± 10.9
**Gender, n(%)**	
*Female*	353(76.9)
*Male*	106(23.1)
**Anthropometric measurements, mean ± SD**	
*Height (cm)*	163.9 ± 8.7
*Weight (kg)*	83.7 ± 14.4
*BMI (kg/m*	31.3 ± 5.1
**Side, n(%)**	
*Right*	237(51.5)
*Left*	223(48.5)
**Number of RF therapy sessions, n(%)**	
*One*	198(43)
*Two*	262(57)

After the RF procedure, the number of patients whose pain decreased according to both AOFAS and Wong-Baker pain scoring systems increased statistically significantly (*p* < 0.001) (Table 2).

**Table 2:** The comparison of the pre- and post-treatment pain status of the patients considering the number of patients

Variables	Pre-treatment	Post-treatment	***P*** values
**AOFAS-pain scores, n(%)**			
*None*	3(0.7)	13(2.8)	**< 0.001**
*Mild, occasional pain*	216(47)	192(41.7)	
*Moderate, daily*	105(22.8)	177(38.5)	
*Severe, almost always present*	136(29.6)	78(17)	
**Wong Baker-pain scores, n(%)**			
*No hurt*	1(0.2)	6(1.3)	**< 0.001**
*Hurts little bit*	27(5.9)	81(17.6)	
*Hurts little more*	73(15.9)	127(27.6)	
*Hurts even more*	207(45)	172(37.4)	
*Hurts whole lot*	122(26.5)	64(13.9)	
*Hurts worst*	30(6.5)	10(2.2)	

McNemar-Bowker test

**Fig. 1:**
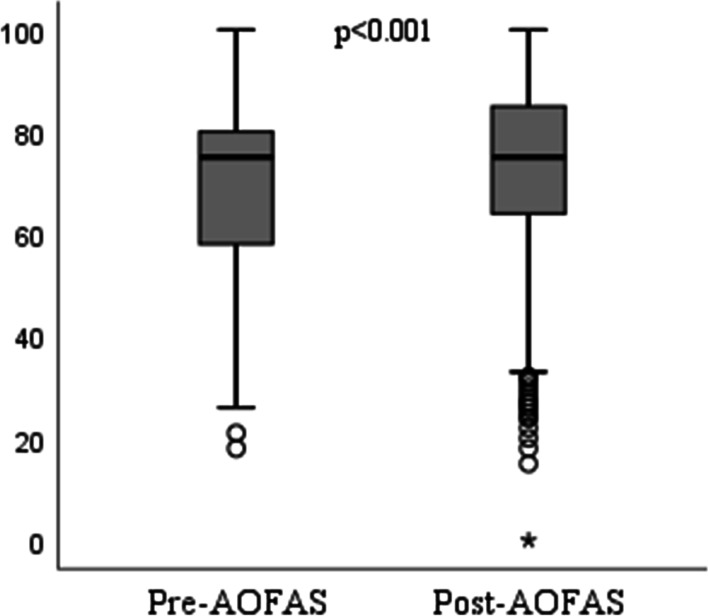
Comparison of pre- and post-treatment AOFAS scores

**Fig. 2:**
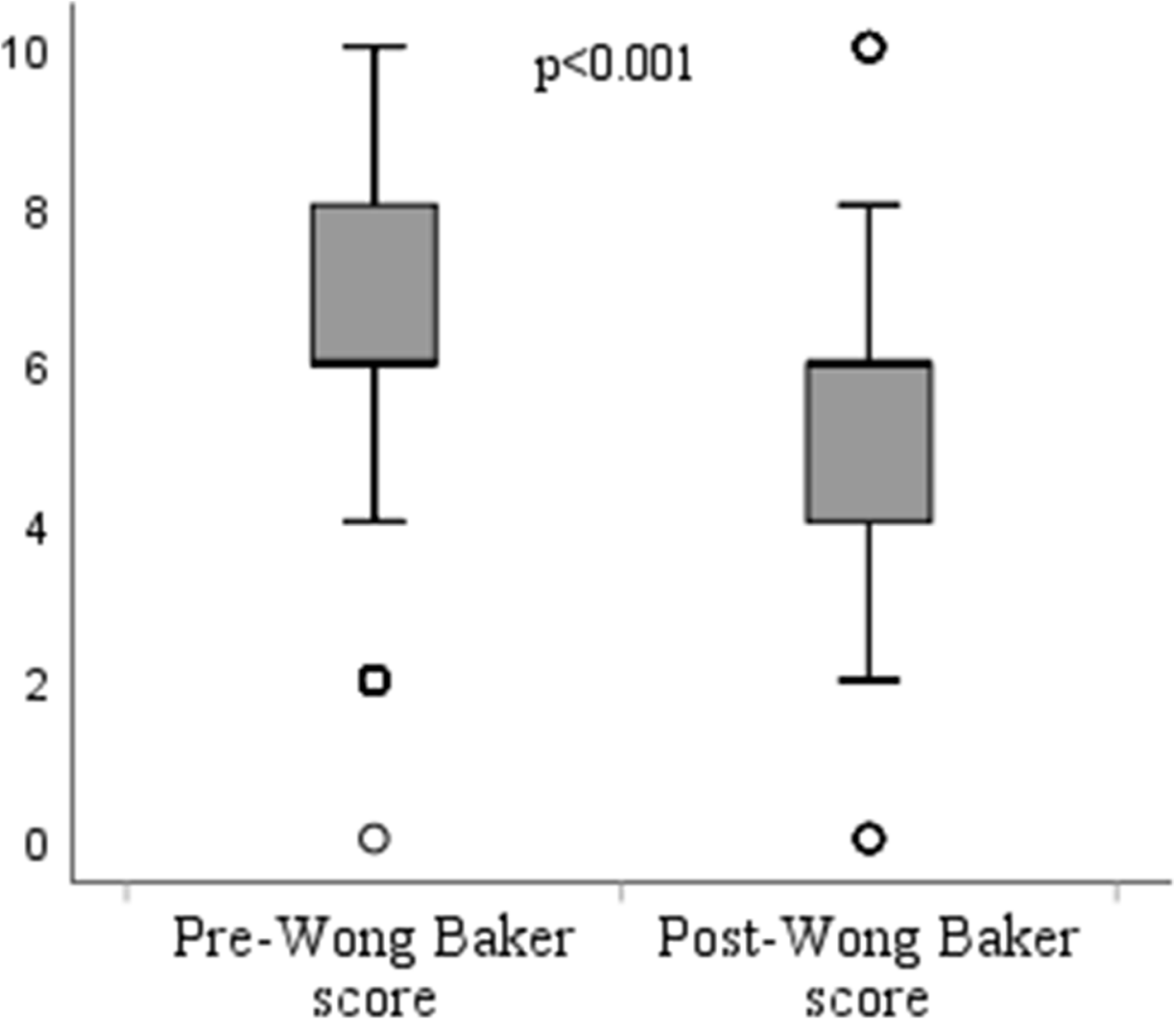
Comparison of pre- and post-treatment Wong-Baker scores

After treatment, a statistically significant increase was found in the AOFAS-pain scores and the total AOFAS scores despite a significant decrease in the Wong Baker-pain score (all *p* < 0.001) (Fig. 1, Fig. 2, Table 3).

**Table 3:** The comparison of pre- and post-treatment clinical and physical examination results of patients considering the scores

Variables	Pre-treatment scores		Post-treatment scores		***P*** value
	Mean ± SD	Median (min-max)	Mean ± SD	Median (min-max)	
**Pain**	16,5 ± 11,5	20(0–40)	21 ± 10,8	20(0–40)	**< 0,001**
**Function**					
Activity limitations, support requirements	8,9 ± 1,9	10(0–10)	8,4 ± 2,2	10(0–10)	**< 0,001**
Maximum walking distance	3,8 ± 1,5	4(0–5)	3,7 ± 1,6	4(0–5)	0,296
Walking surfaces	3 ± 1,7	3(0–5)	3,1 ± 1,6	3(0–5)	0,238
Gait abnormality	6,4 ± 3	8(0–8)	6,4 ± 2,9	8(0–8)	0,901
Sagittal motion	7,3 ± 1,8	8(0–8)	7 ± 2,2	8(0–8)	**0,006**
Hindfoot motion	5,4 ± 1,6	6(0–6)	5,1 ± 1,8	6(0–6)	**0,009**
Ankle-hindfoot stability	7,7 ± 1,4	8(0–8)	7,7 ± 1,5	8(0–8)	0,835
**Alignment**	9,7 ± 1,2	10(0–10)	9,6 ± 1,6	10(0–10)	**0,036**
**Total AOFAS score**	68,7 ± 16,2	75(18–100)	72 ± 17,2	75(0–100)	**< 0,001**
**Wong Baker-pain score**	6,2 ± 1,9	6(0–10)	5 ± 2,1	6(0–10)	**< 0,001**

Wilcoxon Signed Ranks test

The evaluation of the pain status of the patients, who received single-session and double-session RF therapies, by taking the number of sessions into consideration, showed that there was a statistically significant increase in the AOFAS-pain scores and the total AOFAS scores after treatment, as opposed to a significant decrease in the Wong Baker-pain score. However, there was no significant change in treatment success with respect to the number of RF sessions (Table 4).

**Table 4:** The comparison of pre- and post-treatment scores of the patients with respect to the number of sessions

Variables	Single session		Double sessions		***P*** value^**1**^
	Mean ± SD	Median (min-max)	Mean ± SD	Median (min-max)	
**AOFAS-pain scores**					
*Pre-treatment*	15.6 ± 11,5	20(0–40)	17.2 ± 11.5	20(0–40)	0.096
*Post-treatment*	20.4 ± 11,7	20(0–40)	21.5 ± 10.1	20(0–40)	0.484
*p*^*2*^	**< 0.001**		**< 0.001**		
**Total AOFAS score**					
*Pre-treatment*	67.9 ± 15.9	74.5(26–100)	69.3 ± 16.5	75(18–90)	0.226
*Post-treatment*	71.4 ± 18	75(15–100)	72.4 ± 16.5	77(0–100)	0.744
*p*^*2*^	**0.006**		**0.011**		
**Wong Baker-pain scores**					
*Pre-treatment*	6.4 ± 1.9	6(2–10)	6.1 ± 2	6(0–10)	
*Post-treatment*	5.3 ± 2	6(0–10)	4.8 ± 2.1	6(0–10)	
*p*^*2*^	**< 0.001**		**< 0.001**		

^1^Mann-Whitney U test, ^2^Wilcoxon Signed Ranks test

In addition, the patients were evaluated according to the difference in the pre- and post-treatment scores by considering the number of RF sessions applied. Although not statistically significant, the differences in the AOFAS pain scores and in the total AOFAS scores were found to be higher in patients who underwent single-session RF, while the difference in the Wong Baker-pain ranking was higher in patients who received double-session RF (Table 5).

**Table 5:** The comparison of the changes in the pre-treatment and post-treatment scores of the patients with respect to the number of sessions

Variables	Single session		Double sessions		***P*** values
	Mean ± SD	Median (min-max)	Mean ± SD	Median (min-max)	
**Differences in AOFAS-pain scores**	4.8 ± 13.32	0(−30–40)	4.31 ± 13.19	0(−40–40)	0.656
**Differences in total AOFAS scores**	3.52 ± 17.87	2(−44–62)	3.08 ± 17.75	0(−45–60)	0.493
**Differences in Wong Baker-pain scores**	−1.07 ± 2.29	0(−8–6)	−1.29 ± 2.29	−1(−8–4)	0.463

Mann-Whitney U test

When the correlation between BMI and the changes in the post-treatment scores of the patients compared to the pre-treatment (post-treatment-pre-treatment) was examined; It was determined that there was a very weak positive correlation between BMI and the change in pain score (r = 0,114; *p* = 0,018) (Table 6)**.**

**Table 6:** Correlation between BMI and pre- and post-treatment scores

Variables	Pre-treatment		Post-treatment		Difference	
	r	p	r	p	r	p
**Pain**	− 0,112	**0,020**	0,034	0,482	0,114	**0,018**
**Function**						
Activity limitations, support requirements	− 0,055	0,255	− 0,029	0,547	0,008	0,869
Maximum walking distance	−0,105	**0,030**	− 0,097	**0,046**	0,015	0,764
Walking surfaces	−0,094	0,052	−0,095	**0,049**	−0,010	0,839
Gait abnormality	−0,051	0,288	−0,118	**0,015**	−0,075	0,122
Sagittal motion	−0,131	**0,007**	−0,077	0,111	0,014	0,768
Hindfoot motion	−0,121	**0,012**	−0,103	**0,033**	−0,005	0,912
Ankle-hindfoot stability	0,076	0,115	−0,001	0,977	−0,066	0,173
**Alignment**	0,024	0,625	−0,063	0,192	−0,073	0,133
**Total AOFAS score**	−0,140	**0,004**	−0,059	0,223	0,059	0,221
**Wong Baker-pain score**	0,134	**0,005**	0,031	0,522	−0,092	0,058

Spearman correlation test

## Discussion

In this study, following the treatment with intralesional pulsed RF of the patients diagnosed with calcaneal spur, the number of those whose pain decreased according to both the AOFAS Ankle-Hindfoot Score system and the Wong-Baker Faces Pain Rating Scale increased statistically significantly. Moreover, a statistically significant increase was observed after treatment in the pain scores and the total AOFAS scores depending on the AOFAS Ankle-Hindfoot Score system, in contrast to a statistically significant decrease in the Wong-Baker Pain Rating Scale.

The data we obtained regarding the demographic characteristics of patients diagnosed with calcaneal spur in our study, including the mean age being 50.8 ± 10.9, 76.9% of them being female, and the body mass index being 31.3 ± 5.1 kg/m^2^ support the relevant studies in the literature. Also, the fact that no treatment-related complications developed in any of our patients confirms the information that RF therapy is a safe method.

In the literature, there are studies examining the pre-treatment and post-treatment pain and functions of the hindfoot-ankle in patients with chronic heel pain, chronic plantar fasciitis and in a limited number of cases of calcaneal spur, who have been treated with either pulsed or thermal RF treatment applied to intralesional or medial calcaneal / lateral plantar / posterior tibial nerves. However, no study was found in the literature in which intralesional pulsed RF treatment was applied in a large group of patients and in various sessions, for which the results were evaluated.

For example, in the study conducted by Arslan et al., in which they investigated the efficacy of RF neural ablation (RFNA) on 41 feet of 37 patients with chronic heel pain, RFNA was applied to the first branch of the lateral plantar nerve (FBLPN) in one group and to both the FBLPN and medial calcaneal nerve (MCN) in the second group. In both groups, a statistically significant improvement was observed between the pre-procedure visual analog scale (VAS) and those scales observed in the 1st, 6th, and 12th months [16]. Li et al. showed that all 13 patients who underwent arthroscopic RF therapy for painful heel syndrome achieved excellent recovery based on the evaluation made in the 1st and 6th months, and statistically significant improvement was achieved in both VAS and AOFAS ankle-hindfoot scoring [20].

Erken et al. evaluated the 2-year results of RF treatment for the diagnosis of chronic plantar fasciitis by performing percutaneous RF to 35 feet of 29 patients, and showed that there was a statistically significant improvement in the results of VAS and AOFAS scores in the 1st month, 1st year, and 2nd year [21]. In another retrospective study, 22 patients who underwent percutaneous RF nerve ablation treatment for prolonged moderate to severe heel pain associated with plantar fasciitis were evaluated in the 1st week, 1st month, 3rd month and 6th month after treatment. In all those controls, it was observed that the VAS scores of the patients significantly decreased compared to those obtained for the preintervention VAS [22]. Likewise, in the study by Erden et al., in which chronic plantar fasciitis cases were treated, 217 patients were divided into 3 groups according to the treatment methods and evaluated retrospectively, as a result of which it was found that the severity of pain statistically significantly reduced in all patients. It was also shown that the severity of pain decreased significantly more in patients who received corticosteroid injection and RF thermal lesioning in comparison to those who received extracorporeal shock wave therapy [23]. The study of Ayman et al., in which they treated patients with chronic and persistent plantar fasciitis by applying pulsed and thermal RF to the medial calcaneal nerve, reported that the wake-up numerical verbal rating score and the prolonged numerical verbal rating score, which were evaluated in the 1st and 3rd weeks after treatment, resulted in more regression in the pulsed RF group compared to the thermal RF group [15]. The study of Ozan et al., in which patients with plantar fasciitis were treated by applying RF thermal lesioning and extracorporeal shockwave therapy (ESWT), reported that the VAS scores of both groups decreased statistically significantly in the 1st, 3rd and 6th month controls after treatment, while their modified Roley-Maudsley scores decreased statistically significantly just except for the 1st month results of the ESWT group [17].

In the literature, there are studies evaluating the results of the pulsed RF therapy in the treatment of chronic heel pain and chronic plantar fasciitis rather than calcaneal spur. In the treatment of calcaneal spur, the most similar study to ours in terms of applying intralesional pulsed RF is the one conducted by Sır and Eksert with 29 patients with chronic heel pain due to calcaneal spur. In the same study, 15 patients who received intralesional pulsed RF and 14 other patients who received pulsed RF to both intralesional and posterior tibial nerves were compared in terms of hindfoot and ankle pain and their functions before and after treatment. It was shown that numerical verbal rating score (NRS) and AOFAS significantly improved compared to pre-procedure values in both groups at the 3rd week and 3rd month controls. However, no significant difference was found between the groups in terms of NRS and AOFAS [3]. In our study, all patients received only intralesional pulsed RF.

Radiofrequency is successfully used in the treatment of numerous pain conditions such as radicular pain, trigeminal neuralgia, complex regional pain syndrome, sacroiliac joint pain, facet arthropathy, shoulder pain, chronic postsurgical pain, and myofascial pain apart from calcaneal spur. Pulsed RF has been also used successfully in chronic pain conditions for the last 20–25 years [24]. There is evidence for the efficacy of pulsed radiofrequency therapy not only in the treatment of calcaneal spurs, but also in studies with patients with lumbar facet joint pain. For example; Mikeladze et al. reported that more than 50% of 114 patients with cervical, or lumbar facet joint pain undergoing pulsed radiofrequency had a significant reduction in pain [25]. Similarly, Teixeira and Sluijter reported that patients applied pulsed radiofrequency, with low back pain had a significant decrease in pain scores at the end of 3 months [26].

Due to the absence of a patient group in which we could perform neural ablation with the pulsed RF, we were unable to compare two different RF methods, which can be considered as a limitation of our study. Another limitations of our study are; the control evaluation of the patients was made only once and around the 4th week after treatment and the quality of life of the patients before and after the procedure was not evaluated. However, the fact that the data of nearly 500 patients were evaluated and that some patients received double-session RF, thereby enabling us to compare the single-session RF and double-session RF groups, can be shown as the strengths of our study.

## Conclusion

Intralesional pulsed RF procedure can be preferred as a relatively less invasive method that does not have any serious complications in patients with persistent calcaneal spurs who do not respond to the use of oral anti-inflammatory drugs and shoe insoles, nor corticosteroid injection to the lesion area. Further prospective studies with larger patient participation for comparing the treatment results of patients who received intralesional pulsed RF and RF neural ablation may guide clinicians in revealing the advantages and/or disadvantages of intralesional pulsed RF treatment.

## Data Availability

The datasets used and/or analyzed during the current study are available from the corresponding author on reasonable request.

## Abbreviations

AOFAS: American Orthopaedic Foot and Ankle Society
ESWT: Extracorporeal shockwave therapy
FBLPN: First branch of the lateral plantar nerve
RF: Radiofrequency
RFNA: Radiofrequency neural ablation
SD: Standard deviation
VAS: Visual analog scale

## References

[1] Agyekum EK, Ma K (2015). Heel pain: a systematic review. Chin J Traumatol.

[2] Kirkpatrick J, Yassaie O, Mirjailili SA (2017). The plantar calcaneal spur: a review of anatomy, histology, etiology and key associations. J Anat.

[3] Sır E, Eksert S (2019). The use of intralesional and posterior tibial nerve pulsed radiofrequency in the treatment of calcaneal spur. Gulhane Med J.

[4] Atkins D, Crawford F, Edwards J, Lambert M. A systematic review of treatments for the painful heel. Rheumatol. 1999:38(10):968–73. 10.1093/rheumatology/38.10.968 10534547

[5] Krokowska J, Wrona J, Sienkiewicz M, Czernicki J (2016). A comparative analysis of analgesic efficacy of ultrasound and shock wave therapy in the treatment of patients with inflammation of the attachment of the plantar fascia in the course of calcaneal spurs. Arch Orthop Trauma Surg.

[6] Beytemur O, Oncu M (2018). The age dependent change in the incidence of calacaneal spur. Acta Orthop Traumatol Turc.

[7] Banadde BM, Gona O, Vaz R, Ndlovu DM (1992). Calcaneal spurs in a black African population. Foot Ankle.

[8] Toumi H, Davies R, Mazor M (2014). Changes in prevalance of calcaneal spurs in men and women: a random population from a trauma clinic. BMC Musculoscelet Disord.

[9] Riepert T, Drechsler T, Urban R, Schild H, Mattern R (1995). The incidence, age dependence and sex distribution of the calcaneal spur. An analysis of its X-ray morphology in 1027 patients of the central Europian population. RoFo..

[10] Uysal B, Beyzadoglu M, Sager O (2015). Role of radiotherapy in the management of heel spur. Eur J Orthop Surg Traumatol.

[11] Chua NL, Vissers KC, Sluijter ME (2011). Pulsed radiofrequency treatment in interventional pain management: mechanisms and potential indications-a review. Acta Neurochir.

[12] Deniz S, Bakal O, Inangil G. Application of radiofrequency in pain management. Pain management, Milica Prostran. Intech Open. 10.5772/62859 Available from: https://www.intechopan.com/chapters/50353.

[13] Bogduk N (2006). Pulsed radiofrequency. Pain Med.

[14] Cahana A, Zundert JV, Macrea L, van Kleef M, Sluijter M (2006). Pulsed radiofrequency: current clinical and biological literature available. Pain Med.

[15] Osman AM, El-Hammady DH, Kotb MM (2016). Pulsed compared to thermal radiofrequency to the medial calcaneal nerve for management of chronic refractory plantar fasciitis: a prospective comparative study. Pain Physician.

[16] Arslan A, Koca TT, Utkan A, Sevimli R, Akel İ (2016). Treatment of chronic plantar heel pain with radiofrequency neural ablation of the first branch of the lateral plantar nerve and medical calcaneal nerve branches. Foot Ankle Surg.

[17] Ozan F, Koyuncu S, Gurbuz K, Oncel ES, Altay T (2017). Radiofrequency thermal lesioning and extracorporeal shockwave therapy: a comparison of two methods in the treatment of plantar fasciitis. Foot Ankle Spec.

[18] Eyigor C, Eyigor S, Akdeniz S, Uyar M (2015). Effects of intra-articular application of pulsed radiofrequency on pain, fonctioning and quality of life in patients with advanced knee osteoarthritis. J Back Muskuloskeletal Rehabil.

[19] Gulec E, Ozbek H, Pektas S, Isık G (2017). Bipolar versus unipolar intraarticular pulsed radiofrequency thermocoagulation in chronic knee pain treatment: a prospective randomized trial. Pain Physician.

[20] Li SY, Zhang P, Qu F, Wang JL, Liu YJ, Wei M (2013). Arthroscopic treatment of painful heel syndrome with radio-frequency. Zhongguo Gu Shang.

[21] Erken HY, Ayanoglu S, Akmaz I, Erler K, Kiral A (2014). Prospective study of percutaneous radiofrequency nerve ablation for chronic plantar fasciitis. Foot Ankle Int.

[22] Liden B, Simmons M, Landsman AS (2009). A retrospective analysis of 22 patients treated with percutaneous radiofrequency nerve ablation for prolonged moderate to severe heel pain associated with plantar fasciitis. J Foot Ankle Surg.

[23] Erden T, Toker B, Cengiz O, Ince B, Asci S, Toprak A (2021). Outcome of corticosteroid injections, extracorporeal shock wave therapy, and radiofrequency thermal lesioning for chronic plantar fasciitis. Foot Ankle Int.

[24] Byrd D, Mackey S (2008). Pulsed radiofrequency for chronic pain. Curr Pain Headache Rep.

[25] Mikeladze G, Espinal R, Finnegan R (2003). Pulsed radiofrequency application in treatment of chronic zygapophyseal joint pain. Spine J.

[26] Teixeira A, Sluijter ME (2006). Intradiscal high-voltage, long-duration pulsed radiofrequency for discogenic pain: a preliminary report. Pain Med.

